# Estimation of Excess Mortality Rates Among US Assisted Living Residents During the COVID-19 Pandemic

**DOI:** 10.1001/jamanetworkopen.2021.13411

**Published:** 2021-06-14

**Authors:** Kali S. Thomas, Wenhan Zhang, David M. Dosa, Paula Carder, Philip Sloane, Sheryl Zimmerman

**Affiliations:** 1 Center for Gerontology and Healthcare Research, Brown University School of Public Health, Providence, Rhode Island; 2 Center for Long-Term Services and Support, US Department of Veterans Affairs Medical Center, Providence, Rhode Island; 3 Institute on Aging, Portland State University, Portland, Oregon; 4 The Cecil G. Sheps Center for Health Services Research, University of North Carolina at Chapel Hill, Chapel Hill

## Abstract

This cohort study examines rates of mortality among US assisted living residents during the COVID-19 pandemic in comparison with the same period in 2019.

## Introduction

The devastating effects of COVID-19 among older adults residing in long-term care settings have been well documented.^1^ Although much attention has been paid to COVID-19–associated mortality in nursing homes,^2^ less is understood about its effects on assisted living residents. Most assisted living residents are aged 80 years or older and many have multiple chronic illnesses, making them highly susceptible to poor outcomes of COVID-19.^3^ This study examines the excess mortality among a US cohort of assisted living residents during the COVID-19 pandemic.

## Methods

This cohort study was approved by the Brown University institutional review board, which provided a waiver of informed consent because the data are derived from administrative data from the Centers for Medicare & Medicaid Services. This study follows the relevant portions of the Strengthening the Reporting of Observational Studies in Epidemiology (STROBE) reporting guideline.

The study uses data from the Centers for Medicare & Medicaid Services, including the Vital Status data, from 2018 to 2020 (see eMethods in the Supplement for additional details about the data). We applied an established method^4^ relying on 9-digit zip codes to identify a cohort of 425 333 Medicare beneficiaries residing in licensed assisted living settings with 25 or more beds in 49 states and Washington, DC (Minnesota was excluded because it licenses agencies vs physical locations) on January 1, 2019, and 422 262 beneficiaries on January 1, 2020. The primary outcome is the weekly rate of all-cause mortality per 1000 residents. Analyses estimated adjusted incidence rate ratios (aIRRs) and 95% CIs comparing January 1, 2020, through August 11, 2020, with the same weeks in 2019 using Poisson regression with robust standard errors and adjusting for facility fixed effects. We conducted a subgroup analysis limited to 10 states with the highest rate of COVID-19 cases by August 11, 2020 (Rhode Island, South Carolina, New Jersey, Georgia, Alabama, New York, Mississippi, Florida, Arizona, and Louisiana).^5^ Analyses were conducted with Stata statistical software version 16.1 (StataCorp) and SAS statistical software version 9.4 (SAS Institute).

## Results

**Figure 1.**
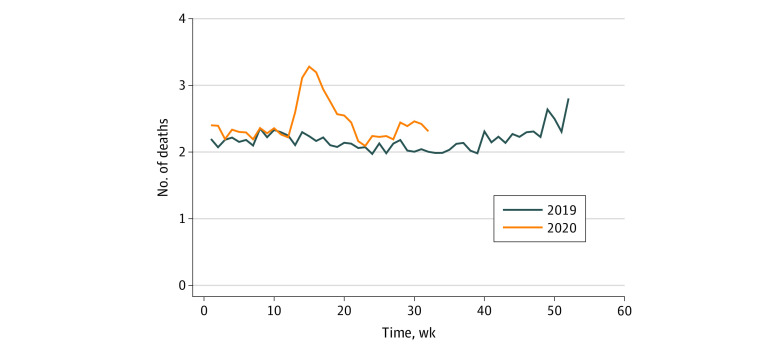
Weekly Observed Deaths per 1000 Medicare Beneficiaries Residing in Assisted Living, 2019 vs 2020 The calendar week begins on January 1 of each year. Cohorts for 2019 and 2020 were identified as having a 9-digit zip code corresponding to a licensed assisted living setting on January 1, 2019 (425 333 participants), and January 1, 2020 (422 262 participants). Deaths represent all-cause mortality and were obtained from the Centers for Medicare & Medicaid Services Vital Status File. Minnesota was excluded because of its different licensing structure. The adjusted incidence rate ratio, comparing 2019 with 2020 mortality rates, is 1.169 (95% CI, 1.142-1.197). The adjusted incidence rate ratio during the peak week (April 8-14, 2020) is 1.359 (95% CI, 1.207-1.529).

**Figure 2.**
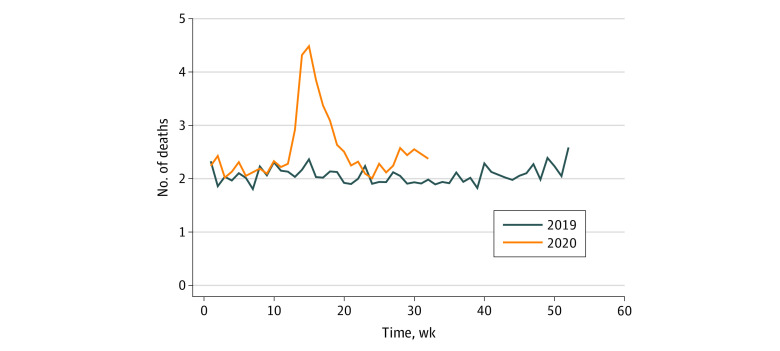
Weekly Observed Deaths per 1000 Medicare Beneficiaries Residing in Assisted Living, Among the 10 States With the Highest Rate of COVID-19 Cases Graph depicts the highest number of COVID-19 confirmed cases per 1 000 000 in the states as of August 11, 2020, as reported by Kaiser Family Foundation’s State Health Facts website.^5^ The 10 states include Rhode Island, South Carolina, New Jersey, Georgia, Alabama, New York, Mississippi, Florida, Arizona, and Louisiana. The adjusted incidence rate ratio, comparing 2019 with 2020 mortality rates is 1.241 (95% CI, 1.185-1.299). The adjusted incidence rate ratio during the peak week (April 8-14, 2020) is 1.728 (95% CI, 1.380-2.163).

In both years, almost one-half of our sample (48%) was older than 85 years, 66% of participants were women, and 90% of participants were White. All-cause mortality rates, nationally, were significantly higher in 2020 compared with 2019 (mean, 2.30 vs 2.02 deaths per 1000 residents per week; aIRR, 1.169; 95% CI, 1.142-1.197; 17% higher overall mortality) (Figure 1). During the peak week in 2020 (April 8 to 14), assisted living resident mortality was 3.28 deaths per 1000 residents per week compared with 2.24 deaths per 1000 residents during the same week in 2019 (aIRR 1.359; 95% CI, 1.207-1.529). New York had the greatest excess mortality between 2020 and 2019 (mean, 2.50 vs 1.57 deaths per 1000 residents per week during January to August) followed by New Jersey (2020 vs 2019, 3.03 vs 2.09 deaths per 1000 residents per week). Among the 10 states with the highest community spread during this period, excess mortality was 2.39 deaths per 1000 residents per week in 2020 during January to August vs 1.93 deaths per 1000 residents per week during January to August in 2019 (aIRR, 1.241; 95% CI, 1.185-1.299; peak week, 4.49 vs 2.37 deaths per 1000 residents per week; aIRR, 1.728; 95% CI, 1.380-2.163; 24% higher mortality) (Figure 2).

## Discussion

After adjusting for facility fixed effects, assisted living residents experienced 17% higher overall mortality in 2020 compared with the year prior (24% higher mortality in the 10 states with the greatest community COVID-19 spread during the study window). These results suggest that assisted living residents experienced increased mortality during the COVID-19 pandemic consistent with increases observed among nursing home residents.^6^

The increase in resident mortality we observed is likely an underestimate of the overall excess mortality during the pandemic given the lag in Vital Status data and the period studied (ie, the analyses were through August 11, 2020, and did not include later surges). Additional limitations include our inability to identify the cause of death and our exclusion of Medicare beneficiaries residing in smaller assisted living settings. Furthermore, we were unable to identify and exclude Medicare beneficiaries who shared a 9-digit residential zip code with a licensed assisted living community but lived elsewhere and residents who relocated after January 1, 2020. Nonetheless, our results provide a first look into the excess mortality during the COVID-19 pandemic among a national cohort of Medicare beneficiaries residing in assisted living.

Future state and federal responses to pandemics targeted to long-term care are advised to explicitly identify the experiences of assisted living settings, recognizing that they differ from nursing homes in terms of overriding model of care (eg, social vs medical model) and staffing (eg, lower staffing levels and less nursing care), among other areas.^3^ In summary, this research calls for specific attention to assisted living in response to pandemics and other emergencies.

## Notes

Supplement. eMethods.
