# Supplementary material for: Estimation of Excess Mortality Rates Among US Assisted Living Residents During the COVID-19 Pandemic
Source: JAMA Netw Open. 2021 Jun 14;4(6):e2113411. doi: 10.1001/jamanetworkopen.2021.13411 (PMC8204200; doi:10.1001/jamanetworkopen.2021.13411)
Supplement: Supplement. — eMethods. [file jamanetwopen-e2113411-s001.pdf]

Supplemental Online Content

Thomas KS, Zhang W, Dosa DM, Carder P, Sloane P, Zimmerman S. Estimation of excess mortality rates among US assisted living residents during the COVID-19 pandemic. *JAMA Netw Open*. 2021;4(6):e2113411. doi:10.1001/jamanetworkopen.2021.13411

eMethods.

This supplemental material has been provided by the authors to give readers additional information about their work.

© 2021 Thomas KS et al. *JAMA Network Open.*

eMethods

<u>Data Sources</u>

Information on assisted living communities comes from a national census we compiled from individual state licensing agencies in 2019. We reviewed state websites and contacted state agents for information on licensed assisted living/residential care settings in each state. Licensing agencies provided information, at a minimum, on the license type, address, and capacity. Following past work, we only include assisted living residences licensed to serve a population of older adults and with capacities for 25+ residents. We exclude the state of Minnesota because of its licensing structure in 2019 (During 2019, Minnesota licensed “home care agencies” to provide assisted living services, rather than the buildings where residents live; therefore, the addresses on which we rely to identify assisted living residents were not available).

The Medicare Master Beneficiary Summary File (MBSF) was used to obtain beneficiaries’ age, race, sex, and dual enrollment in Medicaid. The Vital Status Data were used to derive the date of death. The MBSF was linked to a ZIP Code History File (ZHF) to obtain beneficiaries’ residential ZIP codes and residency starting and ending dates. Medicare Part B Claims (20% file) and the Outcome and Assessment Information Set (OASIS) were used to derive a list of possible ZIP codes that correspond to a large assisted living residence (additional details below) and the Minimum Data Set (MDS) was used to exclude residents who were in a nursing home setting on January 1 of each year.

We used data from the Kaiser Family Foundation’s State Health Facts website to identify rates of community spread. Specifically, we used the data table “COVID-19 and Related State Data” corresponding to our time period (January 1, 2020 - August 11, 2020) to identify the states with the highest COVID-19 case count per 1,000,000 residents.

<u>Identifying a Cohort of Medicare Beneficiaries Residing in AL</u>

We identified Medicare Part B claims in the Carrier File that had a Place of Service code #13 and/or Current Procedural Terminology (CPT) codes 99324-99337, indicating that care was delivered in an assisted living setting. We used the OASIS to identify beneficiaries with a start of care assessment that had a living arrangement code indicating “congregate living situation with around-the-clock assistance.” The Part B Claims/Carrier data and the OASIS were linked to the ZHF to obtain beneficiaries’ 9-digit ZIP codes on the claim and/or OASIS assessment date(s) indicating services were rendered in an assisted living setting. We then compiled the 9-digit ZIP codes of beneficiaries whose first seven digits of their ZIP codes matched to that of an assisted living community’s address. To that list, we added the 9-digit ZIP codes of assisted living communities and created a yearly finder file of 51,231 validated 9-digit ZIP codes associated with 11,617 assisted living communities (many large assisted living

© 2021 Thomas KS et al. *JAMA Network Open.*

campuses have more than one 9-digit ZIP code). Using this finder file, we searched beneficiaries' residential ZIP codes to identify Medicare beneficiaries residing in large assisted living settings with a validated 9-digit ZIP code pertaining to an assisted living community on the last date of prior year. We then used the MDS to exclude beneficiaries who were in a nursing home on January 1, 2019 and January 1, 2020.

© 2021 Thomas KS et al. *JAMA Network Open.*
